# FocaL mass drug Administration for Plasmodium vivax Malaria Elimination (FLAME): study protocol for an open-label cluster randomized controlled trial in Peru

**DOI:** 10.21203/rs.3.rs-5594891/v1

**Published:** 2025-04-17

**Authors:** Sydney Fine, Astrid Altamirano Quiroz, Veronica Soto Calle, Paulo Manrique, Hugo Rodriguez, Gabriel Carrasco, Jade Benjamin-Chung, Adam Bennett, Sarah Auburn, Ric Price, Bryan Greenhouse, J. Kevin Baird, Gonzalo Domingo, Michelle Roh, Angel Rosas, Alejandro Llanos-Cuentas, Michelle Hsiang

**Affiliations:** University of California San Francisco; Universidad Peruana Cayetano Heredia; Universidad Peruana Cayetano Heredia; Harvard University T H Chan School of Public Health; Universidad Peruana Cayetano Heredia; Universidad Peruana Cayetano Heredia; Stanford University; PATH; Menzies School of Health Research: Charles Darwin University; Menzies School of Health Research: Charles Darwin University; University of California San Francisco; Eijkman Institute for Molecular Biology; PATH; University of California San Francisco; Universidad Peruana Cayetano Heredia; Universidad Peruana Cayetano Heredia; University of California San Francisco

**Keywords:** chemoprevention, tafenoquine, primaquine, glucose 6 phosphate dehydrogenase deficiency, cluster randomized control trial, Peru

## Abstract

**Background:**

Outside of sub-Saharan Africa, *Plasmodium vivax* has become the dominant species of malaria. Focal mass drug administration (fMDA) is a potential strategy to support elimination efforts, but controlled studies are lacking.

**Methods:**

The FocaL mass drug Administration for *Plasmodium vivax* Malaria Elimination (FLAME) study is a 3-year cluster randomized controlled trial to determine the impact and safety of fMDA to reduce *P. vivax* transmission. The study will be conducted in Loreto, Peru, where standard interventions have reduced *P. vivax* cases, but transmission persists due to a high proportion of subclinical infections. Thirty low transmission communities (API < 250 cases/1000 population) will be randomized 1:1 to fMDA versus control using a restricted randomization. All communities will receive Peruvian national standard malaria control measures. In the intervention arm, high-risk individuals (living within 200 meters of a *P. vivax* case reported in the prior two years) without contraindication to study medications, including G6PD deficiency, will receive three cycles of fMDA over a two-year period. Each cycle will include two rounds of directly observed therapy delivered 2 months apart. The fMDA regimen will include 25mg/kg chloroquine (CQ) plus a single 300mg dose of tafenoquine (TQ) for individuals age ≥16 years, and 25mg/kg of CQ plus 7 days of 0.5mg/kg/day of primaquine (PQ) if younger. The primary outcome is the cumulative incidence of symptomatic *P. vivax* malaria. The sample size provides 80% power to detect at least a 68% relative reduction in cumulative *P. vivax* incidence, based on alpha of 0.05 and a coefficient of variation (*k*) of 0.87. Secondary outcomes include safety, cost-effectiveness, and infection prevalence and seroprevalence which will be assessed in annual cross-sectional surveys. Safety will be assessed in passive and active pharmacovigilance, including post-treatment screening for G6PD-associated hemolysis by assessing for anemia and hematuria in a sample.

**Discussion:**

The trial will generate evidence regarding fMDA for *P. vivax* and inform malaria elimination efforts in Peru and similarly endemic settings. Findings will be in peer-reviewed publications and through stakeholder meetings in Peruvian and international policy and research forums.

**Trial registration:**

Clinicaltrials.gov NCT05690841. This trial was registered on 09 January 2023.

## BACKGROUND

Current malaria control interventions disproportionally reduce malaria due to *Plasmodium falciparum*, leading to an increase in the proportion of malaria due to *Plasmodium vivax*. Outside of sub-Saharan Africa, *P. vivax* has become the predominant cause of malaria [1–3]. *P. vivax* presents unique challenges for malaria elimination [4]. As with *P. falciparum* malaria, subclinical infections do not come to the attention of the standard health care system. However, unlike *P. falciparum* which invades all red blood cells, *P. vivax* tends to invade reticulocytes, resulting in relatively lower density infections that can be missed by standard diagnostics. Untreated, *P. vivax* persists in its blood stage and remains latent in the liver as a hypnozoite, a stage that reactivates weeks to months later causing symptomatic attacks called relapses [5, 6].

Primaquine, is the only widely available drug that kills *P. vivax* hypnozoites, but its widespread use is undermined by safety, adherence, and efficacy concerns [7]. Primaquine can trigger severe hemolysis in individuals with inherited glucose-6-phosphate-dehydrogenase (G6PD) deficiency.[8] Adherence to the prolonged 7–14 day treatment course is often poor [9], and even complete adherence to standard regimens can lead to recurrent episodes of malaria in approximately 20% of cases [10]. These challenges in case management limit the effectiveness of active case detection approaches [11] and contribute to a reservoir of infections that perpetuate ongoing community transmission.

Mass drug administration (MDA) is a strategy that clears infections that would not otherwise be detected and treated. This approach is focused on the provision of antimalarial treatment to a defined population irrespective of the presence of symptoms or infection often repeated at intervals. In 2015 the World Health Organization (WHO) recommended the use of MDA to reduce *P. falciparum* transmission in very low to low transmission settings, defined as areas where prevalence is less than 10% or incidence is less than 250 cases per 1000 population per year [1, 12], where coverage of standard interventions is high, and risk of importation is low. A recent review of MDA for *P. falciparum* identified eight cluster-randomized controlled trials (CRCT) showing short-term impact [13]. Sustained impact is more likely when baseline transmission intensity is lower, but in these settings where most of the population is not infected and a high proportion of infected individuals have minimal or no symptoms, the risks of adverse events following MDA may outweigh its benefits. Decreased perception of risk can also lead to poor acceptability and adherence [14, 15]. Limiting MDA to the highest risk groups can minimize these risks, while still being effective. Reactive drug administration, or MDA directed at foci of household members and neighbors of index cases was studied in four recent CRCTs that demonstrated it to be effective for reducing community-level transmission of *P. falciparum* [13, 16]. The focal application of MDA to specific higher-risk subpopulations also facilitates higher coverage of MDA, efficient and cost-effective use of limited resources, and safety monitoring [17, 18]. However, the logistical challenges associated with delivery of MDA through this reactive, or “on-call,” approach may preclude its use in many settings.

For *P. vivax*, there are no CRCTs of MDA using radical cure administered either at a village-level or focally. Anecdotal evidence suggests that large-scale mass drug administration to entire communities contributed to *P. vivax* elimination in temperate settings including Azerbaijan, Tajikistan, Afghanistan, North Korea, and China [19, 20]. However, more focal approaches may be an effective strategy that can address the logistical and safety challenges associated with MDA using radical cure – namely the long treatment courses and the potential for G6PD deficiency-associated hemolysis [18]. Most *P. vivax* cases are relapses [21, 22], and relapsing infections can persist for many months and even years. Hence, an obvious strategy would be target individuals harboring hypnozoites [21, 23]. In the absence of a convenient diagnostic test to detect hypnozoites [21, 24], an approach used in Central China was to conduct MDA annually in households of and near index cases reported from the prior 1–2 years. Specifically, focal MDA (fMDA) was conducted annually preceding the high transmission season, which was eventually followed by sustained interruption of *P. vivax* [20]. Operational advantages of this approach are that it self-tailors to changes in transmission levels and the program has time to prepare for delivery that is proactive rather than reactive. Despite lack of controlled data, the WHO made a conditional recommendation for the use of MDA to reduce *P. vivax* transmission given the urgent need for new approaches to eliminate malaria. At the same time, the WHO called for further research on its impact, operational factors, safety, and feasibility, particularly in tropical or subtropical settings [1, 25].

To generate evidence to inform global *P. vivax* elimination efforts, building on the approach used in Central China, we proposed a trial to evaluate the impact and safety of fMDA directed at households in proximity of *P. vivax* index cases from the prior two years. The Loreto region in the Peruvian Amazon was selected as the study site due to its low endemicity of malaria, predominance of *P. vivax*, preliminary data demonstrating cases are primarily subclinical and low density infections, and strong infrastructure to facilitate delivery of fMDA and measure its impact [26]. Importantly, Peru recently approved the use of tafenoquine (TQ), a single dose 8-aminoquinoline which has been shown to be non-inferior to primaquine (PQ) for radical cure, and a new point-of-care test for G6PD deficiency for symptomatic case management. These tools hold promise for facilitating safe and effective delivery of radical cure in communities as part of MDA for *P. vivax* transmission reduction and elimination [27].

### Aims and objectives

The overall study objective is to evaluate the impact and safety of fMDA for *P. vivax* transmission reduction compared to no fMDA. The primary aim is to determine the effect of fMDA on the outcome of cumulative incidence of *P. vivax* cases over the study period. *P. vivax* incidence will be determined based on the number of laboratory-confirmed, locally acquired cases reported from health facilities. Secondary outcomes include incidence of *P. falciparum*, or *P. vivax* and *P. falciparum* and infection prevalence and seroprevalence of *P. vivax* and/or *P. falciparum* measured in cross-sectional surveys. Secondary aims are to assess the safety, tolerability, and acceptability of fMDA, as well as its cost-effectiveness compared to current standard of care interventions. Safety outcomes will be measured through active and passive pharmacovigilance as adverse events and vomiting after administration. Acceptability will be measured as refusal rates and reported willingness to participate in future fMDA campaigns. Cost-effectiveness will be measured as costs per case averted, or per disability adjusted life years (DALY) averted. We hypothesize that fMDA will result in a greatly reduced latent hypnozoite reservoir and that will translate to reduced incidence, infection prevalence, seroprevalence, and cost-effectiveness compared to standard interventions and that it will be safe, well-tolerated, and acceptable to the community.

## METHODS AND DESIGN

**Figure 1:**
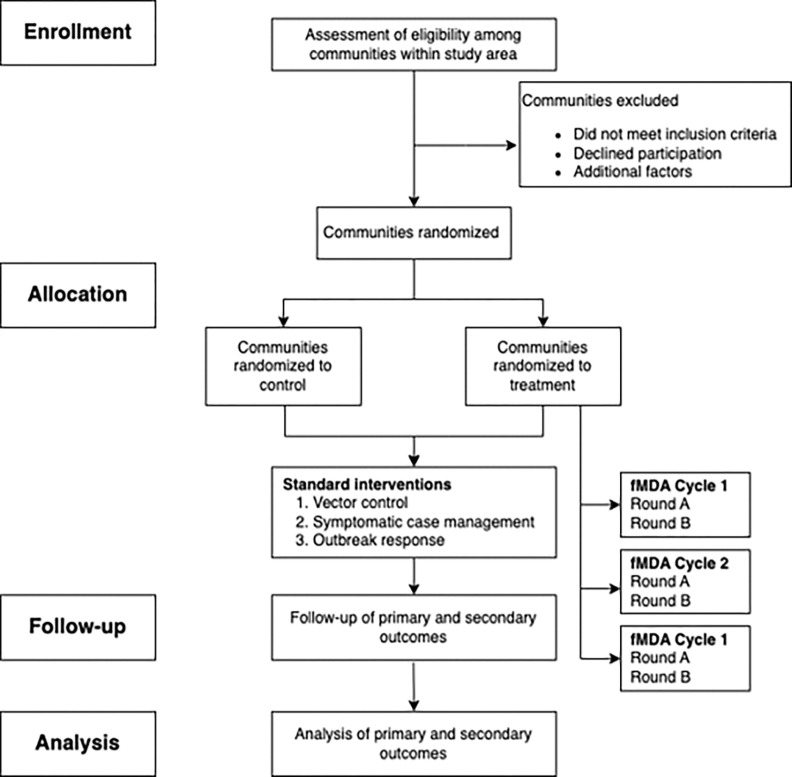
Flowchart demonstrating the involvement of FLAME communities throughout the trial per SPIRIT guidelines. Vector control includes the distribution of insecticide-treated nets (ITNs) and the use of Indoor Residual Spraying (IRR). Symptomatic case management involves testing and treating confirmed malaria cases by microscopy or PCR testing at local health facilities or in the community by a community health worker. Outbreak response may include an active search strategy through screening and treatment of confirmed cases when malaria case incidence is increased significantly compared to the regular trend of cases for two consecutive weeks.

The SPIRIT (Standard Protocol Items: Recommendations for Interventional Trials) recommendations were referenced in developing this protocol [28]. Figure 1 demonstrates the overall study communities’ involvement in the FLAME trial per SPIRIT guidelines.

### Study design

**Figure 2:**
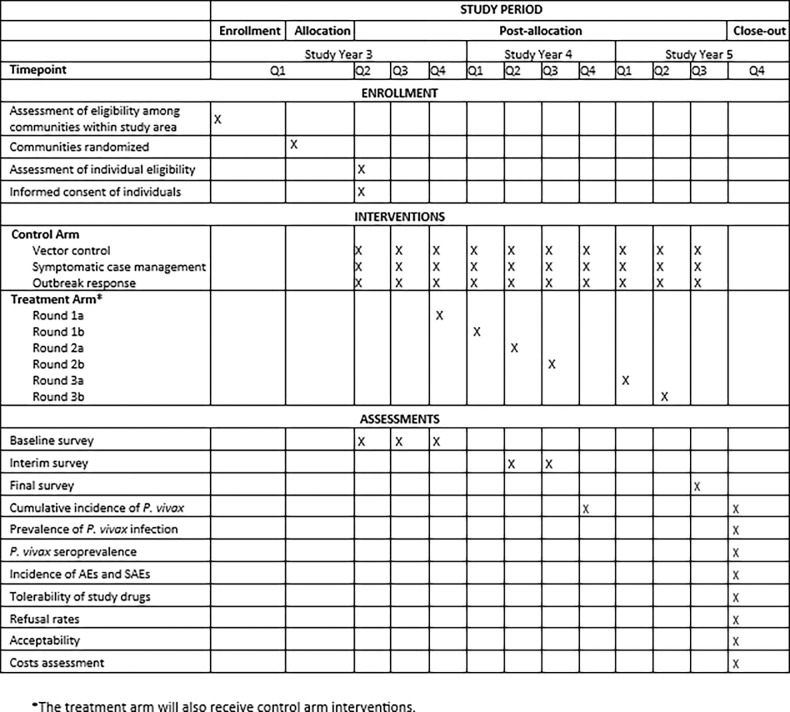
SPIRIT schematic to depict the schedule of enrolment, interventions, and assessments. Study years begin in June and end in May. The treatment arm will receive control arm interventions in addition to the treatment arm interventions.

This is a 3-year open-label CRCT, including enrollment, allocation, post-allocation, and close-out phases (Fig. 2). The trial will be pragmatic and implemented through the existing health system. To demonstrate sustained impact in this tropical setting where strains of *P. vivax* relapse earlier than those once found in Central China [29], the fMDA intervention is relatively aggressive at two rounds per cycle, and the implementation period and follow-up will be longer than prior trials [1].

### Study setting and trial preparations

The trial will take place in Loreto Department, located in the Peruvian Amazon and where more than 90% of the country’s malaria cases occur [30]. Malaria transmission is perennial and historically peaks around April to June, though recently transmission has leveled out throughout the year. Based on national data from the first half of 2024, 82.5% of reported cases were *P. vivax* [30].

**Figure 3:**
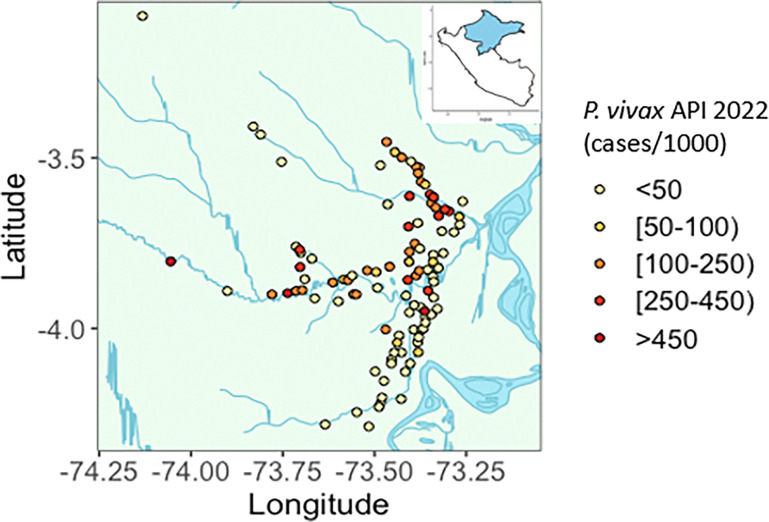
Map of communities within the study area by annual parasite incidence (API) in 2022. API calculated as annual number of malaria cases reported among residents of community per 1000 population. Population data is based on most recent population estimates from 2017 or 2019, depending on the community.

Communities or clusters were eligible if they were located within 8 hours of riverine or road transport of Iquitos (n = 103 communities along the Nanay, Pintuyacu, Itaya, and Monmon Rivers, or along the main road, Carretera Iquitos-Nauta.). Villages with high (API > 250/1000) or sporadic transmission (< 2 cases) in the year prior to the trial, or extreme population size (> 650) were excluded. Utilizing data from 2022, 32 clusters served by 19 health posts met criteria for randomization. Two were eliminated due to transport and budget restrictions. Based on 2023 census data, the population of the 30 communities in the study area is estimated to be 7530. Using case surveillance data from July 2022-June 2023, mean village-level incidence of reported *P. vivax* cases was 65 per 1000 population. A map of the study area and distributions of recent *P. vivax* annual parasite incidences (API) among communities in the region are shown in Fig. 3.

Prior to the trial commencing, activities were conducted to facilitate study implementation and gather baseline epidemiological data. A census and geographic reconnaissance survey was conducted in August 2023 to enumerate the study area and geolocate households. Malaria cases reported between September 2022 to September 2024 were collated from paper-based malaria registers and NOTIWEB, the electronic malaria case reporting system. Based on these and census data, cases in individuals residing in the study area were geolocated to their household.

Community sensitization was undertaken prior to the trial launch and consisted of meeting with Gerencia Regional de Salud (Regional Management of Health) leadership and engaging with local community leaders, health workers, and villagers in meetings. Prior to trial launch, health facilities in both arms received refresher trainings regarding case management and case reporting to bolster the strength of the existing health surveillance program and ensure surveillance data quality across both arms.

### Randomization and Masking

The unit of randomization was a community. A restricted randomization was conducted taking into consideration *P. vivax* incidence in the prior year, distance to a health post, distance to Iquitos, and population size. The Data Manager will remain unblinded to study arm assignment, however, the trial statistician and data analyst will be blinded to the study arm assignment.

### Procedures

#### Enrollment and a baseline assessment

Individual written informed consent will be conducted during the baseline survey or during subsequent surveys for new participants. Verbal consent will be requested separately for each subsequent study procedure, including completion of questionnaires, blood testing, and drug administration. Consent for minors less than 18 years of age will be obtained from a parent or guardian, and minor assent will also be obtained from participants 8–17 years old. Informed consent will be conducted in Spanish.

Enrollment and a baseline assessment in consenting individuals will be conducted prior to the start the trial intervention. At the baseline assessment, demographic, epidemiological, and clinical data will be collected. A finger prick will be conducted for rapid G6PD testing using STANDARD G6PD test (SD Biosensor, Inc., Suwon, South Korea) per manufacturer’s instructions. G6PD status will be categorized as deficient ( ≤ 4.0 IU/g Hb), intermediate (4.1–6.0 IU/g Hb), or normal (≥6.1 IU/g Hb), which reflect ranges of enzymatic activity below or near normal activity of 9.0 IU/g Hb [31], respectively. As the STANDARD G6PD test includes a hemoglobin (Hb) measurement, this result will also be recorded. Blood from the same finger prick will also be used to generate a dried blood spot (DBS) for subsequent malaria molecular testing, as well as subsequent sequencing to identify G6PD variants among G6PD deficient and intermediate individuals, as well as 5% of the G6PD normal individuals. For individuals with fever in the past 48 hours, microscopy will be performed at health establishments, and treatment will be provided per national policy as indicated.

#### Standard malaria interventions in the control and intervention arms

As part of the country’s national malaria elimination program, Plan Malaria Cero (Plan Malaria Zero) [26], standard malaria interventions will be provided in both study arms. These interventions include passive and active case detection using microscopy and treatment per national policy. Current first line treatment is artesunate-mefloquine for *P. falciparum*, and chloroquine (CQ, 10 mg/kg on days 1 and 2, and 5 mg/kg on day 3) plus PQ (0.5 mg/kg/day × 7 days) for *P. vivax* [32]. Tafenoquine (300 mg ×1) is registered in Peru radical cure in individuals that are G6PD normal and ≥ 16 years of age, but only currently available in pilot health facilities outside of the study area. Passive case detection detects symptomatic cases at health facilities. Active case detection up to 6–8 times per year involves village-wide searching for fever, followed by treatment of individuals testing positive by microscopy or rapid test. Vector control interventions include insecticide-treated bed nets provided every 3 years on average and spraying with insecticide approximately 2 times per year.

#### fMDA in the intervention arm

**Figure 4:**
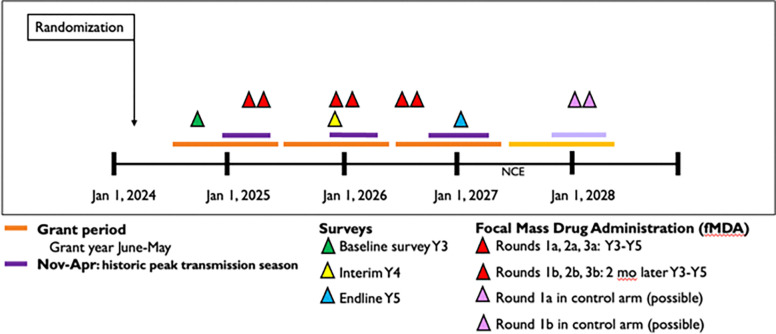
Overall study timeline. Schematic of the study timeline and major activities including surveys and fMDA. Each activity is demarcated with a colored triangle. The grant period includes annual cycles from June-May (orange bars), and the historic peak transmission season includes the period between November-April (purple bars). Abbreviations: NCE= no cost extension.

In the intervention arm, fMDA will be administered over three years in three total cycles, with each cycle including two rounds separated by two months and cycles starting every 9–11 months (Fig. 4). fMDA will be administered by directly observed therapy (DOT) and will target high-risk individuals meeting eligibility criteria detailed in Table 1. These eligibility criteria include being a malaria index case or living in a household within 200 meters of a *P. vivax* index case detected through passive or active case detection and reported in the two years prior to the first round of each fMDA cycle. Individuals with high-risk status will be screened for other inclusion and exclusion criteria for fMDA eligibility.

Each round of fMDA will include CQ plus either TQ, for non-pregnant individuals ≥ 16 years of age and G6PD normal, or PQ, for non-pregnant individuals 6 months to 15 years of age and G6PD normal or intermediate. The prevalence of G6PD deficiency in the study area is anticipated to be <5% [33–37]. Preliminary data from the study area shows that 36.2% of the study population is >6 months and <16 years and will receive PQ. If pediatric TQ is approved for use in Peru during the study, its use will be incorporated into the study.

For the first annual rounds (referred to as 1a, 2a, and 3a for years 1, 2, and 3, respectively) the regimen will include 3 days of CQ (10 mg/kg on days 1 and 2, and 5 mg/kg on day 3) for treatment of *P. vivax* asexual blood stages, with TQ or PQ for *P. vivax* hypnozoite stages (TQ 300 mg × 1, or PQ 5 mg/kg/day × 7 days) (Table 2). PQ and TQ provide prophylactic and gametocytocidal effects against both *P. vivax* and *P. falciparum* [38–42]. However, compared to PQ which has a short half-life of roughly 4–9 hours [43–45], TQ will provide an extended period of protection given its prolonged terminal elimination half-life (~ 15 days), which provides post-treatment prophylaxis for up to 77 days [46, 47].

Coverage and effectiveness of MDA can be compromised by drug eligibility criteria and potential challenges of acceptability, drug efficacy, adherence, imperfect surveillance to identify high-risk individuals, and human movement. Additional rounds of MDA may be needed, particularly in tropical settings where strains are more frequently relapsing. As such, the fMDA intervention will include second annual rounds (referred to as 1b, 2b, and 3b for years 1, 2, and 3, respectively). The regimen will include single-dose CQ (10 mg × 1) with either PQ (5 mg/kg/day × 7 days) or TQ (300 mg × 1, or). CQ, administered as a single dose, will potentiate the anti-relapse effect of PQ, and likely TQ [7, 48, 49]. Administration of this second round at approximately 2 months after each first annual round will serve to prolong the anti-relapse, as well as prophylactic and transmission-blocking activity of fMDA. In multi-site, double-blind, double-dummy, randomized trials comparing TQ to placebo and to PQ [31, 33], efficacy to prevent recurrences was 72.8% (95% CI 65.6–78.8) and 67% (95% CI 61.0–72.3) for PQ and TQ, respectively, at 180 days.

#### Safety

Adverse events (AE) include any unfavorable or unintended sign, symptom, disease, syndrome, abnormal laboratory finding, or illness that emerges or worsens relative to each participant’s pre-treatment baseline, whether or not it is connected to the study intervention. Study clinicians will assess and grade all AEs based on the DAIDS severity grading scale [50]. AEs will be monitored passively, by participant calls to study staff or visits to a local health facility, and actively, through study staff who will actively inquire about AE at monthly visits.

Drug tolerability will be assessed as vomiting or non-adherence due to missed doses. An AE of special interest (AESI) in this study is 8-aminoquionline associated hemolysis which is known to occur in individuals with G6PD deficiency and with malaria itself. Monitoring for this AESI and other adverse reactions will be conducted through passive and active surveillance being conducted in both study arms. Additionally, active pharmacovigilance will occur as study staff inquire about symptoms among during return visits to complete treatment. After receipt of fMDA Round 1a, Hb and urine testing will be conducted on the same day between days 3 and 7 post first treatment with either PQ or TQ [8]. Hb testing will be conducted using the Hemocue portable spectrophotometer per manufacturer’s instructions [8].

For hemolytic events or other severe AEs, participants will be transported within 4 hours to the hospital in Iquitos where blood transfusion therapy and other tertiary care is available. Any participant that has any severe adverse reaction associated with any study drug will be withdrawn from receiving further study drug. The participant will continue to be followed as per protocol except will not receive any additional doses of study medication.

#### Identification of index cases and measurement of Incidence

Malaria case information is recorded in fever books at health posts and these cases are reported to the regional level. Index cases from the 2 years prior to trial will be geo-located prior to each cycle of study medication administration. Index cases also include cases detected during regular interventions by the health system and asymptomatic cases identified during study surveys.

During the trial, for any new or relapse malaria cases diagnosed, a finger prick will be obtained to collect a DBS before administering treatment. A study team member will also conduct a case investigation (within 48 hours of diagnosis) to confirm clinical and demographic details. If a pre-treatment sample was not obtained, study staff will aim to collect a sample from the participant within 3 days.

The primary outcome measure of cumulative incidence is the number of incident *P. vivax* malaria cases divided by the total population. The denominator will include all the participants in the study except for those who have withdrawn from the study.

#### Interim and Endline cross-sectional surveys

An interim survey will be performed prior to the second fMDA cycle, and the final study year will conclude with an endline survey in all clusters. The goal of the surveys will be to consent new participants, update demographic and clinical data, assess acceptability, and collect DBS to assess secondary trial outcomes of infection prevalence and seroprevalence. Acceptability will be assessed both quantitatively and qualitatively, in terms of refusal rates and open-ended questions in the intermediate and endline surveys.

#### Costing and cost-effectiveness assessment

Cost data will be captured in a survey provided to participants who were diagnosed with a confirmed case of malaria by a health establishment. The survey will be administered 15–30 days following the diagnosis to allow potential costs associated with the infection to accrue. Cost data will include direct costs of interventions (e.g., capital expenditures, consumables, personnel, training, transport, and infrastructure), direct costs (e.g., other medications not provided free-of-charge by the interventions, transport costs), and indirect costs from the patient perspective (e.g., lost wages due to malaria per patient and companions).

#### Non-trial care

Participants will be free to seek usual and as-needed medical care at their own discretion, with no effect on study eligibility or arm allocation. Participants undergoing blood testing or receiving medication(s) will receive anticipatory guidance on potential side effects. In the event of symptoms, participants will be instructed to notify the study staff. Study teams encountering individuals with severe or uncomplicated malaria, or other acute illness, will refer such participants to the nearest health facility.

#### Data management

Data will be captured using REDCap forms on electronic tablets and uploaded daily to a secure server. When internet and/or electricity is unreliable, paper forms will be used to collect information instead of tablets. Training on how to capture electronic health data and use the tables will be conducted before each survey and round of fMDA. Study documents will be retained for 10 years following the end of the trial.

#### Laboratory methods

##### Malaria microscopy

Participants will be instructed to seek care at local health posts whenever they have a fever, and malaria testing will be conducted by microscopic blood film examination. Blood smears will be stained with 2% Giemsa for 30 minutes and read by experienced microscopists. Parasite densities will be calculated from the number of asexual parasites per 200 leukocytes (or per 500, if < 10 asexual parasites/200 leukocytes), assuming a leukocyte count of 8,000/μL. A blood smear will be considered negative if examination of 100 high power fields does not reveal asexual parasites. Thin smears will be used for parasite species identification.

##### Molecular methods

Molecular diagnosis by real-time PCR will be performed following the modified protocol reported by Mangold, Manson et al. (2005) [51]. Parasite density calculations will be based on standard curves generated using clinical samples with known parasite concentration. Additionally, more sensitive methods maybe used including amplification of parasitic mitochondrial DNA, which may improve sensitivity more than ten-fold compared to 18s rRNA assays [52, 53]. The amplification of mitochondrial genes could be combined with the use of additional PCR targets to discriminate between species.

Genotyping of both the human and parasite DNA will be performed on blood samples to document G6PD and CYP2D6 genotypes in Peru, to assess within-host parasite genetic diversity, classify infections as local or imported [54], and classify *P. vivax* infections as new, recrudescent, persistent, or relapse [55]. G6PD variants previously associated with G6PD deficiency in Latin America as well as any new variants will be genotyped using long-read sequencing (Oxford Nanopore) of PCR amplicons covering 11 Kb of the ~ 18Kb gene (manuscript in preparation). Similarly, CYP2D6, variants which may impact metabolism and thus efficacy of 8-AQ’s, will be genotyped in a subset of participants with recurrent episodes after fMDA administration. Highly discriminative genotyping will be conducted by Illumina-based targeted deep sequencing of short, variable regions with numerous alleles (microhaplotypes) for *P. falciparum* and *P. vivax* [56–58].

##### Serological methods

Using methods described previously [59, 60], serological tests will be performed using samples from cross-sectional surveys to measure antimalarial antibodies that reflect recent and more distant exposure. Malarial antigens to assess distant exposure may include: *Pv* merozoite surface protein (MSP)-1.19, *Pv* apical membrane antigen (AMA)-1, *Pf* glutamate-rich protein-fragment (GLURP)-R2, and *Pf* AMA-1. Based on preliminary data from our cohort studies, malarial antigens to assess recent exposure may include other objectives such as: *Pv* MSP-10, *Pv* MSP-8, Pv RBP2b [61–63] and *Pf* Early transcribed membrane protein (Etramp)-5. A Gaussian mixture model will be used to determine positivity. Reversible catalytic conversion models fitted by standard maximum likelihood, will be used to generate a seroconversion rate (SCR) [64, 65]. A longitudinal analysis in the same individuals will also enable study of the dynamics in malaria exposure following interventions [66].

## OUTCOMES AND MEASURES

### Sample sizes and power calculations

The study is designed to have 80% power to detect at least 68% relative reduction in cumulative *P. vivax* incidence for fMDA versus control over 3 years, with 15 clusters per study arm (mean population per cluster = 251), based on anticipated baseline *P. vivax* API of 65 cases/1,000 population for the control arm, a coefficient of variation (*k*) of 0.87 based on past data, and a 0.05 two-sided significance level [67]. As Peru is aiming for malaria elimination, this anticipated effect size is considered necessary and consistent with available evidence [18–20, 68, 69].

### Statistical Analysis

#### Primary analysis

An intention-to-treat (ITT) approach will used, in which all clusters with at least one index case during follow-up will be analyzed according to their randomized intervention assignment. For the primary outcome of cumulative incidence of all microscopy-confirmed cases reported in study area residents, negative binomial regression using generalized linear models with cluster-level case counts and cluster population size as an offset will also be used to estimate cumulative incidence ratios (CIR) between study arms [68, 69]. Unadjusted models as well as models adjusted for baseline factors will be fit that are correlated with the outcome (likelihood ratio test p-value < 0.2). Adjusted models may yield higher precision than unadjusted models due to any chance imbalances in baseline covariates. Comparisons of incidence measures will be expressed at the cumulative incidence ratio (CIR) or the protective efficacy (PE = 1-CIR × 100%).

#### Secondary analyses

For secondary outcomes of incidence, the same approach as described above will be used to generate CIRs or adjusted CIRs (aCIRs). For the outcome of clinical malaria, Kaplan-Meier survival curves will be produced. If survival is proportional between study arms, a time to event analysis using Cox proportional regression analysis will be conducted to estimate hazards ratios (HR). For outcomes with continuous variables, a linear regression will be conducted. For prevalence outcomes, log-binomial models will be fit to estimate prevalence ratios (PR). For longitudinally measured outcomes (e.g. parasite prevalence, serology measures, refusal rates), generalized mixed-effect models will be constructed where participant/cluster random effects are included to account for correlation among observations from the same subjects and to account for the clustered study design. Time-intervention interaction will be evaluated to assess difference in trend between arms. Key subgroup analyses such as age, sex, baseline transmission intensity, distance to Iquitos, distance to a health post, and population size will be performed.

Per-protocol analyses will restrict to clusters in which at least 80% of interventions were delivered according to the study protocol. Analyses will use g-methods to separately adjust for baseline and post-treatment covariates associated with non-compliance [70].

#### Spillover effects

An effective intervention must decrease or interrupt transmission among individuals receiving the intervention (a “direct effect”) as well as among non-recipients outside of intervention zones (a “spillover effect”) [71–73]. There is a biological basis for spillover effects of fMDA. Chemotherapy can block transmission by reducing gametocyte biomass, and subsequent movement of humans and mosquitos can result in spillover effects outside of intervention zones. Our primary analysis at the cluster level is a pooled effect estimate across intervention recipients and non-recipients. Direct and spillover effects of fMDA will be estimated by comparing malaria incidence and prevalence in non-recipients in proximity to fMDA zones by arm. Direct effects will be defined as the cumulative incidence ratio (or prevalence ratio) among treated individuals in focal treatment zones within 200m of index case in the intervention arm vs. the control arm. Spillover effects will be defined as the cumulative incidence ratio among treated individuals outside focal treatment zones around index cases in the intervention arm vs. the control arm.

#### Safety and tolerability analysis

Serious adverse events refer to any expected or unexpected event, related or unrelated to the study medication, that results in death, a life-threatening event, hospitalization, prolongation of hospitalization, disability or incapacity, or a congenital anomaly. The incidence of serious adverse events (SAE) from fMDA, defined as SAEs divided by the product number of participants that received study drug and time, will be measured. Sub-analyses will be conducted by drug type. The incidence of SAE or severe malaria in fMDA will also be compared to the incidence of severe malaria in the control arm [74–77]. AEs and adherence will also be assessed descriptively.

Tolerability will be assessed as vomiting following administration of study drugs and non-adherence due to missed doses. Adverse event monitoring will be conducted both actively and passively.

#### Cost-effectiveness

A cost-effectiveness analysis will be conducted from both provider and patient perspectives. Health outcomes for participants allocated to fMDA and control interventions will be estimated using probabilistic decision tree models, and then compared to determine the incremental effects of fMDA in terms of *P. vivax* incident and prevalent infection averted and disability-adjusted life years (DALY) averted. Results of the trial will be the main source of data on probabilities and complemented with data from the malaria surveillance systems and the scientific literature. DALYs will be estimated using disability weights and life expectancies at death from the WHO life tables. Cost data will include direct and indirect costs of interventions. Costs will be adjusted for inflation and to reflect local government salaries. Deterministic and probabilistic sensitivity analyses will be conducted to understand the effect of parameter uncertainty (effect probabilities and costs) on the incremental cost-effectiveness ratio (ICER) of fMDA, expressed as the cost per *P. vivax* incident infection averted and cost per DALY averted. The analysis will be conducted at the end of the trial after all rounds of fMDA have been completed.

#### Ethics

The study will be conducted in accordance with accepted principles on Ethics in Human Experimentation and ICH/GCP. Participants will be compensated a total of 30 soles in cash per family per visit to compensate them for their time they contribute to the clinical trial away from their jobs. This money will be paid in cash and will be recorded in a document signed by the head of each family acknowledging their compensation. In order to protect the privacy of patients, only authorized study personnel will have access to paper records, and those records will be kept in a locked file at the site facility, Asociación Civil Selva Amazónica. Later they will be transported to the Malaria and Leishmania Unit at the IMTAvH—UPCH in Lima in compliance with established requirements (Reglamento de Ensayos Clínicos). No names will appear on any forms or publications. No information will be shared with anyone else outside of the study without participants’ permission.

#### Monitoring and auditing

The DSMB will be included in discussions prior to the start of the trial to review the analysis plan and throughout the trial to advise on interim analyses. Should early evidence of intervention safety problems arise, the DSMB will advise on stopping the study. The study will be halted if > 11/1,000 individuals have incidence of any SAE, including by not limited to AHA, related to taking an 8-aminoquinoline.

Both an internal monitor and an external monitor will oversee the clinical trial. The external monitoring agency will conduct regular site visits including to the laboratory. The internal monitor will help develop a detailed monitoring plan including when/how to review patient charts, who will conduct monitoring visits, and who will address findings from monitoring.

The FLAME study team holds regular meetings throughout to communicate about study progress and procedures. The primary study personnel from both UPCH and UCSF met weekly to plan and track trial activities. The study team will meet monthly to review incoming data as it becomes available. The entire study team holds meetings approximately quarterly.

## DISCUSSION

### Potential challenges

A key challenge that may be encountered is ensuring wide coverage of the intervention. Community members may be gone for days at a time working in the field, sleep in multiple homes, or live most of the time in the city of Iquitos. To maximize coverage of fMDA, medications will be delivered house to house by DOT at times convenient to the participant. Villagers that miss the first round of fMDA in a cycle or become eligible between the first round and second round will be re-considered for fMDA eligibility prior to the subsequent cycle.

Capturing all incident cases of malaria is also challenging. Participants may not seek care when they feel ill or may have an asymptomatic malaria infection. In order to capture as many cases as possible, households will be visited on a monthly basis by the field team to inquire about symptom onset or case diagnosis. DBS samples will be collected from all individuals at two timepoints which may aid in the detection of subclinical infections. Participants will be encouraged to seek care if they feel ill during the study.

The study communities in which we will intervene are dynamic and mobile in nature. The platform QField will be used to update maps of the study communities each visit and participants will be asked during monthly community vigilance if any individuals in their home are new or have left. In this way, population estimates will be maintained as accurately as possible.

## TRIAL STATUS

Recruitment of study participants began on 14 October 2024 utilizing Spanish protocol version 5.6 from 9 September 2024. Initial enrollment efforts will be completed in March 2025; however, participants may be enrolled throughout the trial until the completion of the trial in May 2026.

## Figures and Tables

**Table 1.** Eligibility criteria for study activities

	Inclusion Criteria	Exclusion Criteria
Clusters	· Within 8 hours transport of Iquitos · Incidence <250/1000 and >2 cases in the year prior to trial · Population size (<650)	· Outside of study area including villages on the banks of the Momon, Nanay, Itaya, and Pintuyacu rivers and along the main road in the region, Carretera Iquitos-Nauta
Study Enrollment	· Slept in household within study community at least one night in the past four weeks · 6 months old · Signed informed consent	
fMDA		
Chloroquine	· Resides in neighboring household but within 200 m of Pv index case in the past 2 years · Age ≥6 months old · Present for intervention · Adult ≥18 years old that provides informed consent · A child ≥8 years and <18 years old that provides informed assent and has informed consent from their parents · A child ≥6 months old and <8 years old that has informed consent from their parents	· History of retinal or visual field changes · Known hypersensitivity or adverse reaction to CQ · Currently taking CQ or have taken CQ in the past four weeks · Ineligible for TQ or PQ (see criteria below) · Hemoglobin <9 g/dL
Tafenoquine	· Eligible to receive CQ · Age ≥16 years old · Adult ≥18 years old that provides informed consent · A child ≥16 years and <18 years old that provides informed assent and has informed consent from their parents	· G6PD deficiency or intermediate status (defined as activity 6.0 UI/gHb per SD biosensor) · G6PD status unknown or refusal of G6PD status test · Acute or severe malaria · Pregnancy (known or identified by pregnancy test) · Refusal of pregnancy test if new amenorrhea in the past 4 weeks · Woman breastfeeding a child that is G6PD deficient or with unknown G6PD status · Known hypersensitivity or adverse reaction to TQ or PQ · Have taken mefloquine (i.e. artesunate-mefloquine), TQ or PQ, or other antimalarial in the past four weeks · Hemoglobin < 9 g/dL
Primaquine	· Eligible to receive CQ and ineligible to receive TQ · Age ≥6 months old · Adult ≥18 years old that provides informed consent · A child ≥8 years and <18 years old that provides informed assent and has informed consent from their parents · A child ≥6 months old and <8 years old that has informed consent from their parents	· G6PD deficiency (defined as activity <4.0 UI/gHb per SD biosensor) · G6PD status unknown or refusal of G6PD status test · Acute or severe malaria · Pregnancy (known or identified by pregnancy test) · Refusal of pregnancy test if new amenorrhea in the past 4 weeks · Breastfeeding child with documented or unknown G6PD deficiency status · Known hypersensitivity or adverse reaction to TQ or PQ · Have taken mefloquine (i.e. artesunate-mefloquine), TQ or PQ, or other antimalarial in the past four weeks · Hemoglobin < 9 g/dL

**Table 2.** Drugs formulations and doses for all fMDA Rounds

Drug*	Dosage form (mg)	Administration	Manufacturer	Dose	
				≥16 years	6 mos – 15 years
**Chloroquine (CQ)**					
3-day CQ (Rounds 1a, 2a, 3a)	150 mg base	Take with food to minimize possible gastrointestinal irritation	Kronos Lab, Guayaquil, Ecuador	Dose ~10 mg/kg (D1, D2), ~5 mg/kg (D3)	Dose ~10 mg/kg (D1, D2), ~5 mg/kg (D3)
sdCQ (Rounds 1b, 2b, 3b)				Dose ~10 mg/kg once	Dose ~10 mg/kg once
**Tafenoquine (TQ)** (all Rounds)	150 mg	TQ should be taken with food to increase absorption	Piramal Pharma Ltd, Mumbai, India	300 mg once	n/a
**Primaquine (PQ)** (all Rounds)	15 mg	Take with food	Colompack S.A., Bogota, Colombia	Dose ~0.5 mg/kg daily x 7 days (If TQ is contraindicated)	Dose ~0.5 mg/kg daily x 7 days

fMDA: focal mass drug administration; CQ: chloroquine; sdCQ: single dose chloroquine; TQ: tafenoquine; PQ: primaquine; D1: Day 1; D2: Day 2; D3: Day 3

* The commercial name of the product and the manufacturer may vary depending on if the product is bought or donated.

## Abbreviations

CQ: Chloroquine
CRCT: Cluster-randomized control trial
DALY: Disability-adjusted life year
DOT: Directly observed therapy
FLAME: Focal mass drug administration for vivax malaria elimination
fMDA: Focal mass drug administration
G6PD: Glucose-6-phosphate dehydrogenase
Hb: Hemoglobin
MDA: Mass drug administration
NCE: No-cost extension
PQ: Primaquine
Pv: Plasmodium vivax
TQ: Tafenoquine
WHO: World Health Organization
